# Effect of acetylcholinesterase inhibitors on post-stroke cognitive impairment and vascular dementia: A meta-analysis

**DOI:** 10.1371/journal.pone.0227820

**Published:** 2020-02-07

**Authors:** Jin Ok Kim, Soo Joo Lee, Jung-Soo Pyo

**Affiliations:** 1 Department of Neurology, Eulji University Hospital, Eulji University School of Medicine, Daejeon, Republic of Korea; 2 Study group for meta-analysis, Eulji University Hospital, Eulji University School of Medicine, Daejeon, Republic of Korea; 3 Department of Pathology, Eulji University Hospital, Eulji University School of Medicine, Daejeon, Republic of Korea; Nathan S Kline Institute, UNITED STATES

## Abstract

Cognitive impairment is a common complication observed after a stroke. Currently there are no definitively proven pharmacologic therapies for recovery from post-stroke cognitive impairment and vascular dementia. In this meta-analysis, we evaluated the efficacy and safety of cholinesterase inhibitors in their improvement of cognition in patients with post-stroke cognitive impairment and vascular dementia. We conducted a meta-analysis using seven eligible studies from 305 published articles. We investigated the differences in Mini–Mental State Examination (MMSE) and Alzheimer’s Disease Assessment Scale-Cognitive subscale (ADAS-Cog) scores, before and after cholinergic augmentation in patients with post-stroke cognitive impairment and vascular dementia. MMSE and ADAS-cog scores were also compared during the subsequent follow-up periods. MMSE score of patients with post-stroke cognitive impairment was increased after cholinergic augmentation throughout the 24 weeks with mean differences [MD] of 3.000, 1.732, 1.578 1.516, and 1.222, at 4, 4–8, 8–12, 12–18, and 18–24 weeks, respectively. In addition, ADAS-cog scores decreased at 6, 12, 18, and 24 weeks by pharmaceutical augmentation, but not with placebo with mean differences [MD] of -2.333, -2.913, -2.767, -2.416, and -1.859, respectively. This meta-analysis shows that acetylcholinesterase inhibitors maintain a stable pattern of improved cognitive function in patients with post stroke cognitive impairment and vascular dementia without the increased risk of side effects.

## Introduction

Post-stroke cognitive impairment is a common complication observed after stroke. The prevalence of dementia within the first year after stroke ranges from 9% to 30% [1]. However, the prevalence of cognitive impairment with no dementia may be much higher. Post-stroke cognitive impairment can also be a risk factor for vascular dementia. The prevalence of new-onset dementia shortly after a first incidence of stroke is about 10% after a recurrent stroke excluding pre-stroke dementia is about 30% [2]. Stroke itself is one of the main causes of vascular dementia after a stroke [2].

Recovery after stroke arises spontaneously and may last weeks, even years especially for the recovery of language skills and cognition [3]. After 1 year, only 10% of the stroke patients with cognitive impairment with no dementia recover [4]. Pharmacotherapy accelerates spontaneous recovery of post-stroke cognitive impairment, and enhancement of cognition might further facilitate functional recovery. Pharmacological interventions modulating stroke-induced disruption of diverse neurotransmitters may further enhance cognition through brain plasticity and long-term strengthening [5].

Cholinergic agents, such as donepezil, rivastigmine, and galantamine, are commonly used to treat vascular dementia. While evidence exists from large randomized controlled trials on the efficacy of these cholinergic agents in the treatment of Alzheimer’s dementia, their efficacy in the treatment of post stroke cognitive impairment remains less defined. A meta-analysis by Kavirajan and Schneider found that cholinesterase inhibitors might produce small benefits in cognition in patients with mild to moderate vascular dementia; however, this evidence was not significant enough for widespread use of acetylcholinesterase inhibitors [6]. Furthermore, recent drug trials reported that cholinergic boosting using donepezil had a beneficial effect in chronic stroke patients with aphasia after a 16-weeks treatment regimen [7]. Another pilot study suggested that donepezil might lead to improved functional recovery in the immediate post-stroke period [8]. We therefore aimed to evaluate the efficacy and adverse effects of these pharmacological interventions in the treatment of post-stroke cognitive impairment and vascular dementia.

## Materials and methods

### Published study search and selection criteria

Relevant articles were obtained by searching the PubMed and MEDLINE databases for studies published prior to November 15, 2019. The database was searched using the following key words and search string: ‘Stroke’ AND ‘cholinesterase inhibitors’ OR ‘donepezil’ OR ‘rivastigmine’ OR ‘galantamine’. The titles and the abstracts of all searched articles were screened including review articles in order to find additional eligible studies. The search results were then reviewed and included if (1) the study was performed on human patients, and (2) there was information for the MMSE or ADAS-cog scores after cholinergic augmentation in patients with infarction or vascular dementia. Case reports or non-original articles, and non-English language publications were excluded.

### Data extraction

Data from all eligible studies were extracted by two independent authors. The data extracted from each of the eligible studies included; the paper reference, first author’s name, year of publication, study location, regimen of pharmaceutical augmentation, number and age of the patients, MMSE, ADAS-cog scores, or Clinician’s Interview-Based Impression of Change-Plus (CIBIC-Plus), and any complications that may have risen after cholinergic augmentation or placebo treatment. In addition, the assessment for the quality of nonrandomized studies in the meta-analysis was performed using the Newcastle-Ottawa Scale.

### Statistical analyses

All data were analyzed using the Comprehensive Meta-Analysis software package (Biostat, Englewood, NJ). We analyzed the mean differences of MMSE and ADAS-cog scores, before and after cholinergic augmentation in patients with post-stroke cognitive impairment and vascular dementia. The change of MMSE and ADAS-cog scores during various follow-up periods were also compared. Complications such as minor or severe adverse effects, and deaths were also noted. Since the eligible studies included different populations, a random-effects model was deemed more suitable than a fixed-effects model. Heterogeneity between the studies was checked using the Q and *I*^2^ statistics, and presented using *P*-values. Sensitivity analysis was conducted to assess the heterogeneity of eligible studies and the impact of each study on the combined effect. Additionally, the meta-regression test was performed in order to elucidate the heterogeneity between subgroups. To assess for publication bias, Begg’s funnel plot and Egger’s test were used. The results were considered statistically significant at *P* < 0.05.

## Results

### Selection and characteristics

**Fig 1:**
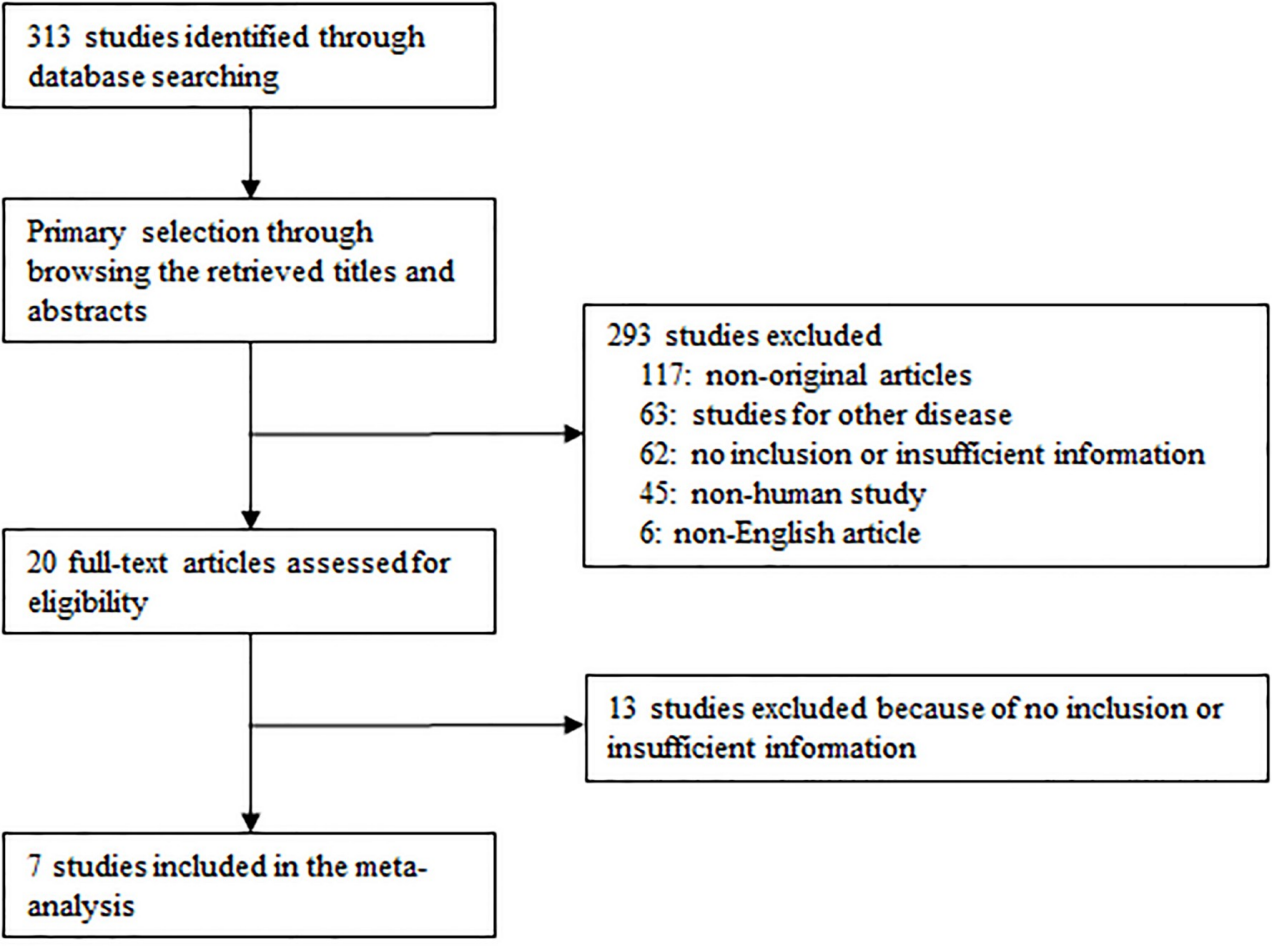
Flow of information through the different phases of the eligible studies.

We searched the PubMed database using the before mentioned keywords (see Material and methods) and identified 305 reports. Among them, 117 articles were excluded because of non-original articles, 63 articles for insufficient information and 63 articles due to their study of other diseases, and further 51 articles because they used animals or cell lines (n = 43) and not written in English (n = 6). The remaining 7 studies were included in the meta-analysis (Fig 1 and Table 1) [9–15].

**Table 1:** Main characteristics of the eligible studies.

Study	Location	Subgroup	Regimen	Number of patients	Male	Female	Age (Mean)	MMSE (Mean±SD)	ADAS-cog (Mean±SD)	CIBIC-Plus (Mean±SD)
Black 2003	UK	VD	Placebo	199	115	84	74.2	21.7±0.3	20.1±0.7	
			Donepezil 5mg/day	198	111	87	73.7	21.9±0.3	21.2±0.8	
			Donepezil 10mg/day	206	107	99	73.9	21.8±0.3	20.9±0.7	
Chang 2011	Korea	PSCI	Placebo	4	2	2	55 (28–74)	24.8 (24–26)		
			Donepezil	6	4	2	55.5 (45–69)	24.2 (23–36)		
Moretti 2008	Italy	PSCI	Rivastigmine	50	19	31	74.23	18.6±2.1		
		VD	Rivastigmine	50	24	26	73.23	20.7±2.0		
Narasimhalu 2010	Singapore	PSCI	Placebo	25	12	13	69.4 (48–84)	23.9±3.2	30.4±14.1	
			Rivastigmine	25	5	20	68.1 (57–81)	23.7±3.4	29.9±13.1	
Pratt 2002	Various	VD	Placebo	392	220	172	74.3 (41–91)	21.6±4.2	22.3±11.1	
Roman GC 2010	US	VD	Placebo	326	176	150	72.3	23.6±0.3	21.7±0.6	3.6±0.05
			Donepezil	648	398	250	73.4	23.5±0.2	21.8±0.4	3.6±0.03
Wilkinson 2003	Various	VD	Placebo	193	105	88	74.4	22.2±0.3	18.8±0.7	
			Donepezil 5mg/day	208	130	78	74.7	21.8±0.3	20.8±0.7	
			Donepezil 10mg/day	2115	134	81	75.7	21.5±0.3	20.6±0.7	

Numbers in parentheses represent percentage

Abbreviations: MMSE, mini–mental state examination; ADAS-cog, Alzheimer’s Disease Assessment Scale-Cognitive subscale; CIBIC-Plus, Clinician’s Interview-Based Impression of Change-Plus; UK, United Kingdom; VD, vascular dementia; PSCI, post-stroke cognitive impairment; US, United States

### Meta-analysis for the correlation between MMSE or ADAS-cog score/acetylcholinesterase inhibitor

**Fig 2:**
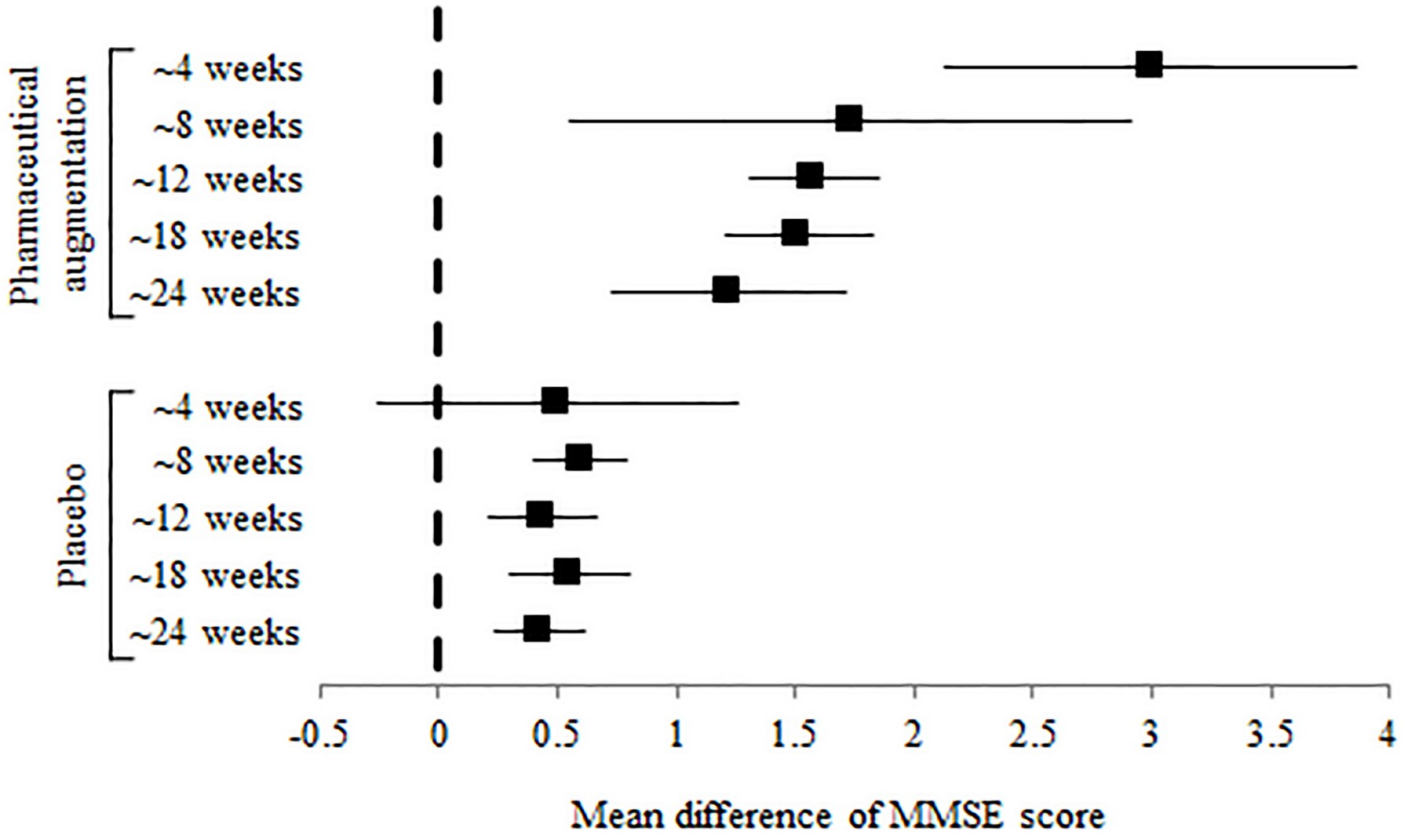
The mean difference of MMSE(mini–mental state examination) score according to the pharmaceutical augmentation.

Mean differences (MD) of MMSE score in post-stroke cognitive impairment and vascular dementia with cholinergic augmentation were significantly increased throughout the 24 weeks: 3.000 (95% confidence interval [CI] 2.135 to 3.865) at 4 weeks, 1.732 (95% CI 0.555 to 2.910) at 4–8 weeks, 1.578 (95% CI 1.308 to 1.848) at 8–12 weeks, 1.516 (95% CI 1.203 to 1.829) at 12–18 weeks, and 1.222 (95% CI 0.727 to 1.718) at 18–24 weeks (Table 2 and Fig 2). Only minimal change of MMSE score was observed compared to placebo in post-stroke cognitive impairment and vascular dementia after 4 weeks (Table 2 and Fig 2). In addition, there was no significant impact of each study on estimated values.

**Table 2:** The mean difference of MMSE score according to the pharmaceutical augmentation.

	Number of Subsets	Heterogeneity test [*P*-value]	Random effect [95% Confidence interval]	Egger’s Test [*P*-value]
in 4 weeks				
Placebo	1	1.000	0.500 [-0.259, 1.259]	-
Pharmaceutical augmentation	1	1.000	3.000 [2.135, 3.865]	-
4–8 weeks				
Placebo	3	0.913	0.600 [0.403, 0.796]	0.870
Pharmaceutical augmentation	3	< 0.001	1.732 [0.555, 2.910]	0.107
Donepezil 5mg/day	2	< 0.001	2.208 [-0.466, 4.882]	-
Donepezil 10mg/day	1	1.000	0.968 [0.588, 1.347]	-
8–12 weeks				
Placebo	2	0.446	0.437 [0.206, 0.668]	-
Pharmaceutical augmentation	2	0.300	1.578 [1.308, 1.848]	-
Donepezil 5mg/day	1	1.000	1.387 [0.938, 1.836]	-
Donepezil 10mg/day	1	1.000	1.677 [1.361, 1.994]	-
12–18 weeks				
Placebo	2	0.742	0.552 [0.302, 0.801]	-
Pharmaceutical augmentation	2	0.686	1.516 [1.203, 1.829]	-
Donepezil 5mg/day	1	1.000	1.581 [1.138, 2.023]	-
Donepezil 10mg/day	1	1.000	1.452 [1.009, 1.894]	-
18–24 weeks				
Placebo	4	0.434	0.421 [0.233, 0.610]	0.901
Pharmaceutical augmentation	5	< 0.001	1.222 [0.727, 1.718]	0.331
Donepezil 5mg/day	2	0.778	1.113 [0.652, 1.575]	-
Donepezil 10mg/day	2	0.688	1.589 [1.270, 1.908]	-

Abbreviations: MMSE, mini–mental state examination

**Fig 3:**
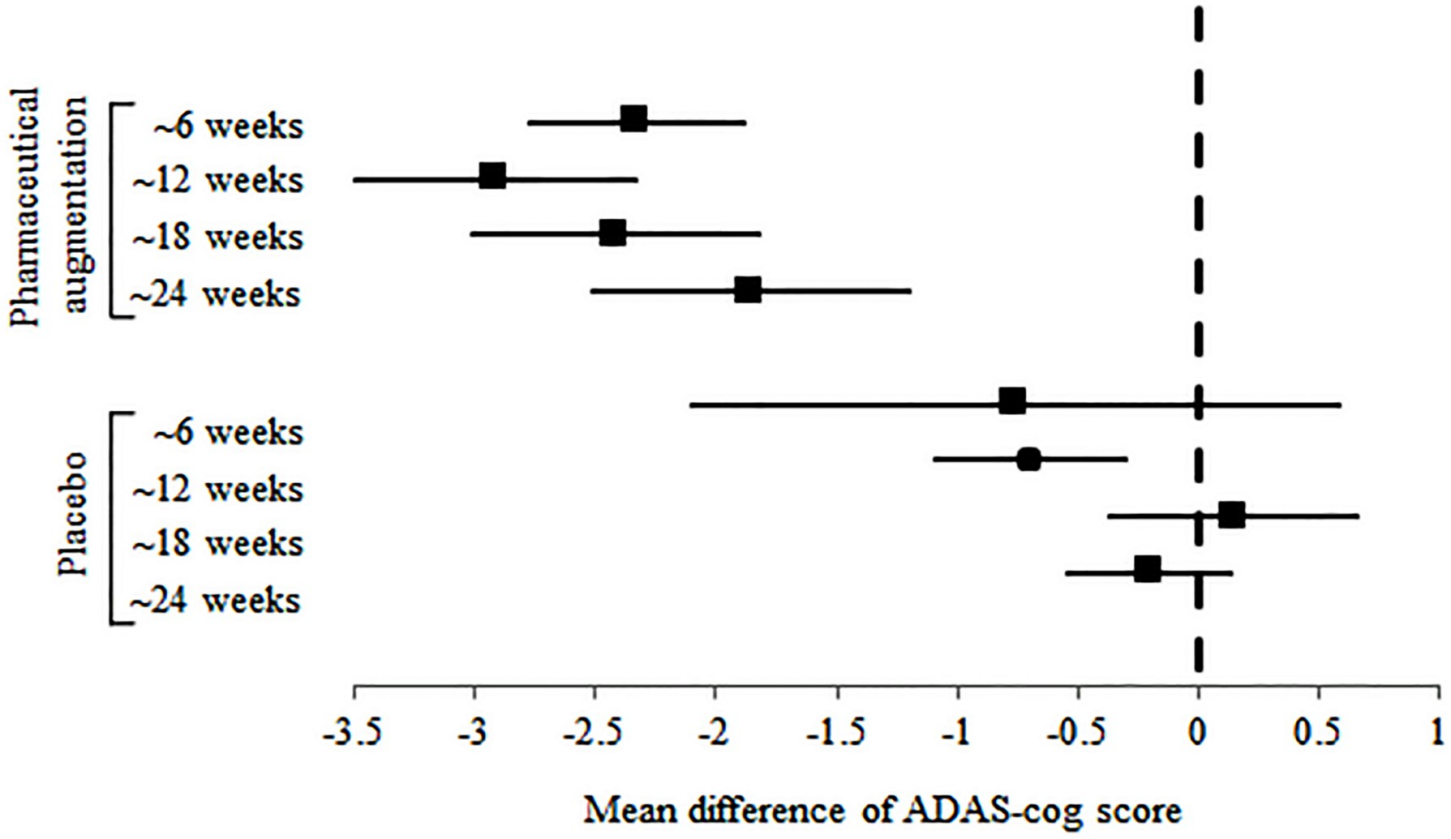
The mean difference of ADAS-cog score according to the pharmaceutical augmentation.

Next, the changes of ADAS-cog scores were evaluated with and without cholinergic augmentation. No studies were found comparing ADAS-cog in patients with post stroke cognitive impairment, and only the ADAS-cog score of patients with vascular dementia was comparable. ADAS-cog scores by cholinergic augmentation were found to be decreased by: MD of -2.333 (95% CI -2.778 to -1.889) at 6 weeks, MD of -2.913 (95% CI -3.490 to -2.335) at 12 weeks, MD of -2.416 (95% CI -3.009 to -1.824) at 18 weeks, and MD of -1.859 (95% CI -2.514 to -1.204) at 24 weeks and this decreased pattern was maintained for 24 weeks (Table 3 and Fig 3). In patients with vascular dementia, however, no effects of placebo on ADAS-cog scores were observed at follow-ups at 6, 18, and 24 weeks (MD -0.763, 95% CI -2.104 to 0.578; MD 0.148, 95% CI -0.365 to 0.133; and MD -0.204, 95% CI -0.541 to 0.133, respectively), but were observed at 12 weeks (MD -0.699, 95% CI -1.092 to -0.307).

**Table 3:** The mean difference of ADAS-cog score according to the pharmaceutical augmentation.

	Number of Subsets	Heterogeneity test [*P*-value]	Random effect [95% CI]	Egger’s Test [*P*-value]
6 weeks				
Placebo	2	< 0.001	-0.763 [-2.104, 0.578]	-
Pharmaceutical augmentation	2	1.000	-2.333 [-2.778, -1.889]	-
Donepezil 5mg/day	1	1.000	-2.333 [-3.013, -1.654]	-
Donepezil 10mg/day	1	1.000	-2.333 [-2.921, -1.745]	-
12 weeks				
Placebo	2	0.384	-0.699 [-1.092, -0.307]	-
Pharmaceutical augmentation	2	0.542	-2.913 [-3.490, -2.335]	-
Donepezil 5mg/day	1	1.000	-2.767 [-3.511, -2.022]	-
Donepezil 10mg/day	1	1.000	-3.133 [-4.048, -2.219]	-
18 weeks				
Placebo	2	0.197	0.148 [-0.365, 0.661]	-
Pharmaceutical augmentation	2	0.471	-2.416 [-3.009, -1.824]	-
Donepezil 5mg/day	1	1.000	-2.267 [-2.985, -1.548]	-
Donepezil 10mg/day	1	1.000	-2.733 [-3.779, -1.688]	-
24 weeks				
Placebo	4	0.292	-0.204 [-0.541, 0.133]	0.775
Pharmaceutical augmentation	5	0.002	-1.859 [-2.514, -1.204]	0.081
Donepezil 5mg/day	2	0.265	-1.924 [-2.507, -1.342]	-
Donepezil 10mg/day	2	0.212	-2.386 [-3.179, -1.592]	-

Abbreviations: ADAS-cog, alzheimer’s disease assessment scale-cognitive subscale;,CI, confidence interval

The improved rate in CIBIC-Plus was significantly higher in patients with donepezil 5mg/day than placebo group (*P* < 0.001 in a meta-regression test). However, there was no significant difference of improved rate in CIBIC-Plus between Donepezil 10mg/day and placebo subgroup (*P* = 0.693 in a meta-regression test). We added the methods and results for CIBIC-Plus in the revised manuscript.

Adverse effects were observed in 0.669 (95% CI 0.269 to 0.917) of patients with placebo and 0.813 (95% CI 0.516 to 0.946) of patients with cholinergic augmentation (Table 4). Severe adverse effects were found in 0.145 (95% CI 0.100 to 0.206) and 0.138 (95% CI 0.119 to 0.160) of patients with placebo and cholinergic augmentation, respectively. Death occurred in 0.018 (95% CI 0.010 to 0.032) and 0.009 (95% CI 0.000 to 0.189) in patients with cholinergic augmentation and placebo, respectively.

**Table 4:** The adverse effects according to the pharmaceutical augmentation.

	Number of Subsets	Heterogeneity test [*P*-value]	Random effect [95% CI]	Egger’s Test [*P*-value]
Adverse effect				
Placebo	4	< 0.001	0.669 [0.269, 0.917]	0.426
Pharmaceutical augmentation	6	< 0.001	0.813 [0.516, 0.946]	0.055
Severe adverse effect				
Placebo	3	0.099	0.145 [0.100, 0.206]	0.666
Pharmaceutical augmentation	4	0.541	0.138 [0.119, 0.160]	0.075
Death				
Placebo	2	0.058	0.009 [0.000, 0.189]	-
Pharmaceutical augmentation	2	0.408	0.018 [0.010, 0.032]	-

Abbreviations: CI, confidence interval

## Discussion

Significant improvements of MMSE score were found between cholinergic augmentation and placebo groups in patients at 4 weeks with post stroke cognitive impairment and vascular dementia. ADAS-cog scores in patients with vascular dementia improved with cholinesterase inhibitor treatment and maintained a stable pattern of improved cognitive function compared to the placebo group through the 24 weeks. A study showed that ADAS-Cog improvement is likely clinically meaningful, whereas many patients with no change in the ADAS-Cog still show meaningful improvement [16]. In addition, the intensity and direction of the initial treatment response appear to be important in informing long-term outcomes [16].

Cholinergic pathways and the neural system are vulnerable to vascular damage and can lead to cognitive impairment. The cholinergic system includes the basal forebrain, substantia innominate, striatum, cerebral cortex (mainly pyramidal neurons and medium-sized neurons in layers III and V of the motor and secondary sensory areas), some brainstem nuclei, and spinal motor neurons [17]. The entire cerebral cortex and the white matter contains a dense network of cholinergic fibers originating from the nucleus basalis of Meynert [18]. Acetylcholinesterase inhibitors have been known to modulate cognitive function by compensating for the lack of intracerebral cholinergic neurotransmitters through inhibition of acetylcholine hydrolysis which is an effective treatment pathway in patients with post-stroke cognitive impairment and vascular dementia [19]. The mechanisms by which acetylcholinesterase inhibitors promote recovery from post stroke cognitive impairment are currently not fully understood, but two mechanisms are mostly considered to be: 1) Acetylcholine acting as a cortical modulator playing a critical role in practice-related plasticity [20] and, 2) the regulation of cerebral circulation influenced by cholinergic mechanisms and acetylcholine improving regional cerebral blood flow in vascular dementia patients [17]. In support of the first mechanism, experimental studies in rodents have shown that cholinergic fibers are associated with structural adaptation and functional recovery, thereby highlighting a key role for acetylcholine in rehabilitation-mediated recovery from traumatic brain injury [21]. For the latter, acetylcholinesterase inhibitor might restore the impaired cognition by improving on mechanisms that couple neuronal activity and vascular status of the brain.

Placebo-treated patients with post stroke cognitive impairment displayed minor improvements on MMSE after 4 weeks and only marginal improvements on ADAS-cog at 12 weeks (Tables 2 and 3). In general, post stroke cognitive impairment and motor dysfunction show recovery during the subacute phase and reach a plateau or level off after several months following the onset [22–23]. Patient with post-stroke cognitive impairment does not worsen without the recurrence of a stroke. As the deterioration of the cognitive function continues, there is a possibility that the pathology of the degenerative disease also plays a role. Our results also indicate that the ADAS-cog score is generally unchanged with the only change being significant at 12 weeks.

Adverse effects and severe adverse effects were near significant in both placebo and cholinergic augmentation groups. Death in the cholinergic augmentation groups was two-fold, but the total number of deaths was very small in both groups. It is therefore not a necessary concern as to when a patient with a stroke undergoes cholinergic augmentation in order to avoid severe adverse effects and a possible death. A recent study of 33 patients found that donepezil is safe when initiated within the 24 hours of a stroke onset [24]., thereby confirming that administration of acetylcholinesterase inhibitor after an acute stroke is safe and well tolerated.

This meta-analysis has some limitations. There are no detailed indicators that can be used to establish risk and benefit from the different patterns and severity of cognitive impairment after a stroke. First, the total number of trials included in the analysis was very small, with only two trials on cholinergic enhancement in patients with cognitive impairment after a stroke. Therefore, our meta-analysis can be more meaningful. The other trials were on cholinergic enhancement in patients with vascular dementia. Second, vascular dementia is heterogeneous because it groups together a broad category of patients with a variety of cerebrovascular diseases that include impaired cognitive function. In these five clinical trials, 61% to 70% of patients with vascular dementia experienced more than one transient ischemic attack or stroke, suggesting that there were a significant number of patients affected with post stroke cognitive impairment. However, the information of patients with post stroke cognitive impairment could not be obtained from included studies. Third, only ADAS-Cog scores and MMSE score changes were compared in the present meta-analysis. However, other evaluating methods for cognitive status of patients could not find from included studies. Fifth, the detailed information for the effect of short term treatment could not be obtained due to too low number of included studies.

In conclusion, our analysis of placebo-controlled studies suggests that acetylcholinesterase inhibitors are helpful for vascular dementia and post-stroke cognitive impairment. We demonstrate that cholinergic augmentation is well tolerated in patients with cognitive impairment and vascular dementia. Further longitudinal studies with a longer follow-up assessment are vital to further evaluate the efficacy of cholinesterase inhibitors in the early treatment of post-stroke cognitive impairment.

## Supporting information

S1 Checklist PRISMA 2009 checklist. (DOC)

S1 File Assessment of the Newcastle-Ottawa Scale of eligible studies. (DOCX)
