# Supplementary material for: Effect of acetylcholinesterase inhibitors on post-stroke cognitive impairment and vascular dementia: A meta-analysis
Source: PLoS One. 2020 Feb 7;15(2):e0227820. doi: 10.1371/journal.pone.0227820 (PMC7006920; doi:10.1371/journal.pone.0227820)
Supplement: S1 File — (DOCX) [file pone.0227820.s002.docx]

| Study | Location | Subgroup | Regimen | Number  of patients | MMSE  (Mean±SD) | ADAS-cog  (Mean±SD) | CIBIC-Plus  (Mean±SD) |
| --- | --- | --- | --- | --- | --- | --- | --- |
| Black 2003 | UK | VD | Placebo | 199 | 21.7±0.3 | 20.1±0.7 |  |
|  |  |  | Donepezil 5mg/day | 198 | 21.9±0.3 | 21.2±0.8 |  |
|  |  |  | Donepezil 10mg/day | 206 | 21.8±0.3 | 20.9±0.7 |  |
| Chang 2011 | Korea | PSCI | Placebo | 4 | 24.8 (24-26) |  |  |
|  |  |  | Donepezil | 6 | 24.2 (23-36) |  |  |
| Moretti 2008 | Italy | PSCI | Rivastigmine | 50 | 18.6±2.1 |  |  |
|  |  | VD | Rivastigmine | 50 | 20.7±2.0 |  |  |
| Narasimhalu 2010 | Singapore | PSCI | Placebo | 25 | 23.9±3.2 | 30.4±14.1 |  |
|  |  |  | Rivastigmine | 25 | 23.7±3.4 | 29.9±13.1 |  |
| Pratt 2002 | Various | VD | Placebo | 392 | 21.6±4.2 | 22.3±11.1 |  |
| Roman GC 2010 | US | VD | Placebo | 326 | 23.6±0.3 | 21.7±0.6 | 3.6±0.05 |
|  |  |  | Donepezil | 648 | 23.5±0.2 | 21.8±0.4 | 3.6±0.03 |
| Wilkinson 2003 | Various | VD | Placebo | 193 | 22.2±0.3 | 18.8±0.7 |  |
|  |  |  | Donepezil 5mg/day | 208 | 21.8±0.3 | 20.8±0.7 |  |
|  |  |  | Donepezil 10mg/day | 2115 | 21.5±0.3 | 20.6±0.7 |  |
| Numbers in parentheses represent percentage  Abbreviations: MMSE, mini–mental state examination; ADAS-cog, Alzheimer's Disease Assessment Scale-Cognitive subscale; CIBIC-Plus, Clinician’s Interview-Based Impression of Change-Plus; UK, United Kingdom; VD, vascular dementia; PSCI, post-stroke cognitive impairment; US, United States | | | | | | | |
